# Advances in nuclear medicine-based molecular imaging in head and neck squamous cell carcinoma

**DOI:** 10.1186/s12967-022-03559-5

**Published:** 2022-08-12

**Authors:** Danni Li, Xuran Li, Jun Zhao, Fei Tan

**Affiliations:** 1 Shanghai Fourth People’s Hospital, and School of Medicine, Tongji University, Shanghai, China; 2 Department of Nuclear Medicine, Shanghai East Hospital, School of Medicine, Tongji University, Shanghai, China; 3 The Royal College of Surgeons in Ireland, Dublin, Ireland; 4 The Royal College of Surgeons of England, London, UK

**Keywords:** Head and neck squamous cell carcinoma, Molecular imaging, Nuclear medicine, Epidermal growth factor receptor, Somatostatin receptor, Cancer-associated fibroblasts, Programmed cell death ligand 1, Prostate-specific membrane antigen

## Abstract

Head and neck squamous cell carcinomas (HNSCCs) are often aggressive, making advanced disease very difficult to treat using contemporary modalities, such as surgery, radiation therapy, and chemotherapy. However, targeted therapy, e.g., cetuximab, an epidermal growth factor receptor inhibitor, has demonstrated survival benefit in HNSCC patients with locoregional failure or distant metastasis. Molecular imaging aims at various biomarkers used in targeted therapy, and nuclear medicine-based molecular imaging is a real-time and non-invasive modality with the potential to identify tumor in an earlier and more treatable stage, before anatomic-based imaging reveals diseases. The objective of this comprehensive review is to summarize recent advances in nuclear medicine-based molecular imaging for HNSCC focusing on several commonly radiolabeled biomarkers. The preclinical and clinical applications of these candidate imaging strategies are divided into three categories: those targeting tumor cells, tumor microenvironment, and tumor angiogenesis. This review endeavors to expand the knowledge of molecular biology of HNSCC and help realizing diagnostic potential of molecular imaging in clinical nuclear medicine.

## Introduction

### Head and neck cancer and its treatment

Head and neck cancer (HNC) is the seventh most common cancer globally, accounting for 2–4% of all cancers worldwide. The incidence of HNC is about 890,000 new cases yearly [1]. HNC includes a variety of tumors arising from the mucosal surface of several major anatomical sites in the upper aerodigestive tract: the oral cavity, sinonasal cavity, pharynx (nasopharynx, oropharynx and hypopharynx), and larynx. Ninety percent of HNCs are squamous cell carcinoma (SCC) [2]. The main risk factors associated with head and neck squamous cell carcinoma (HNSCC) include, but are not limited to, heavy tobacco and alcohol consumption, human papillomavirus (HPV) infection or Epstein-Barr virus (EBV) infection [3]. Currently, the primary treatment modalities for HNSCC are surgery, radiotherapy and chemotherapy, with targeted therapy and immunotherapy as emerging oncotherapies. Despite the above treatments, the five-year survival rate of advanced HNSCC patients remains poor around 40–50% without significant improvement over the past several decades [4, 5]. In order to improve the prognosis of these patients, both early diagnosis and effective treatment are crucial. Therefore, there has been constant need for developing novel and enhancing existing diagnostic approach for HNSCC.

### Targeted therapy for HNSCC

As a form of molecular medicine, targeted therapy is a cancer treatment that uses drugs to target specific genes or proteins that control cancer cells growth, division, and spread, without affecting normal cells. Biomarkers for targeted therapy can be applied to predict response to specific therapy, predict response regardless of therapy, or to monitor response once a therapy has initiated [6]. So far, there has been several clinically available medications targeting chosen biomarkers for HNSCC treatment with promising outcome. For example, cetuximab, an epidermal growth factor receptor (EGFR) inhibitor, is used to treat recurrent or metastatic HNSCC. However, the indication for this agent is relatively narrow, and the treatment response rate remains suboptimal [7, 8]. In this context, early prediction of treatment sensitivity or resistance to this targeted therapy may spare patients from futile treatment cycles and unnecessary side effects.

### Molecular imaging for targeted therapy

Due to the marked heterogeneity of biomarker expression in primary and metastatic tumors, the current biopsy and immunohistochemistry (IHC) methods routinely used in clinical practice are not entirely accurate or comprehensive in assessing the true expression of biomarkers. The anatomic and volumetric imaging, such as computed tomography (CT) and magnetic resonance imaging (MRI), also has limitations. In contrast, molecular imaging uses molecular probes that specifically bind to targets, generating or amplifying detectable signals for direct visualization and quantification of biomarker expression in vivo. This dynamic and quantitative characterization allows early identification of patients who may benefit from targeted treatments, monitoring of treatment efficacy and assessing potential toxicity [9, 10]. Molecular imaging promotes targeted therapy by predicting therapeutic effect, evaluating early response, and determining therapeutic regimen.

### Nuclear medicine-based molecular imaging and its potential for HNSCC patients

Among the available molecular imaging modalities, nuclear medicine-based ones are most sensitive in detecting biomarker expression. Nuclear medicine-based molecular imaging can detect and assess multiple lesions simultaneously, allowing for repetitive and non-invasive evaluation. The nuclear medicine imaging helps recognize the biological behavior of malignant tumors at the molecular level, aiming to clarify tumor-specific information, such as changes in blood flow, metabolism, receptor density and function, abnormal gene expression and cellular information transmission. For example, fluorine-18 fluorodeoxyglucose (^18^F-FDG) positron emission tomography (PET) scan coupled with CT or MRI has been widely used in HNSCC diagnosis and tumor staging, especially for locoregional and/or distant metastasis [11]. In addition to ^18^F-FDG, extensive researches have been conducted to develop novel imaging agents for diagnostic, therapeutic and prognostic assessment.

**Fig. 1:**
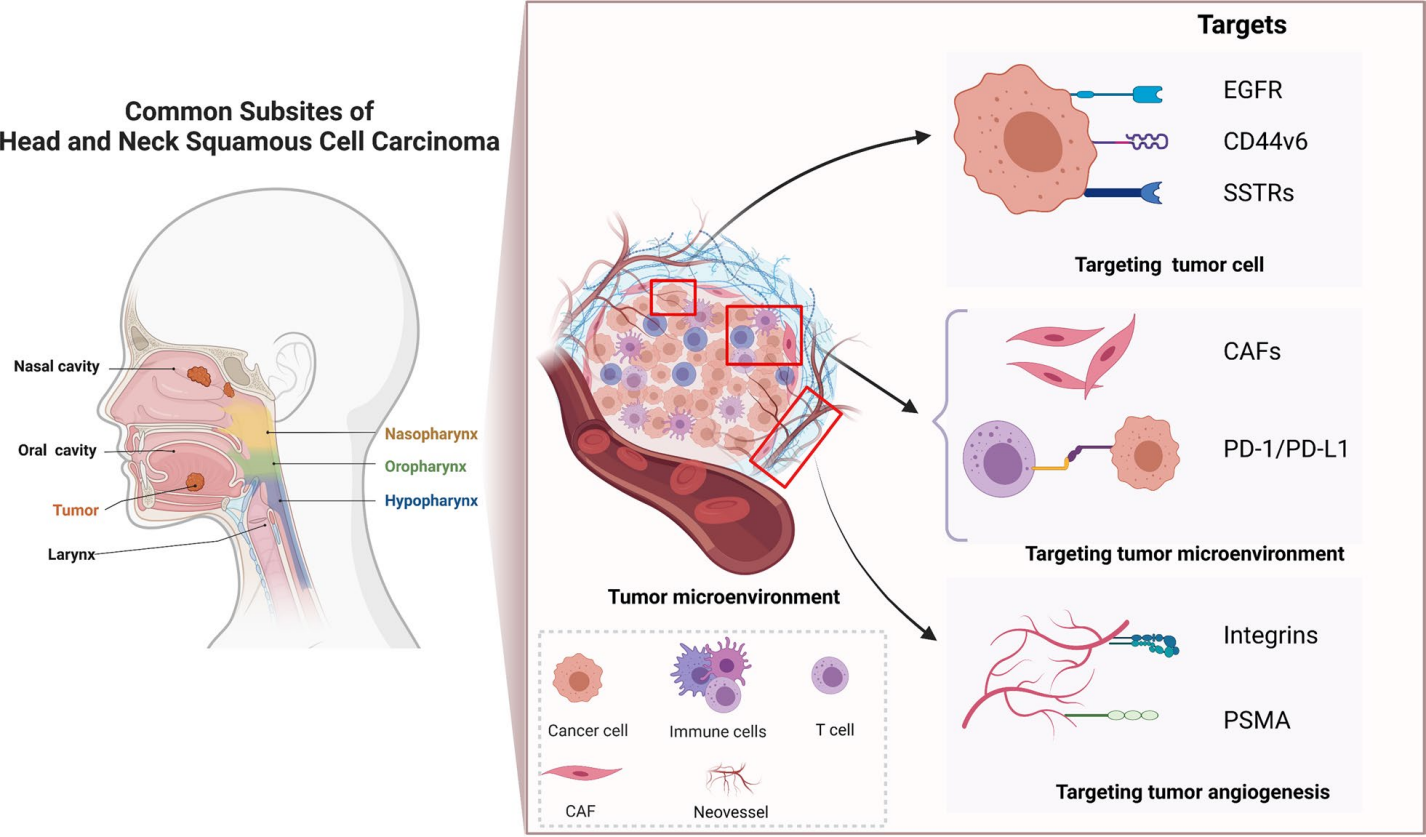
Schematic diagram showing the common subsites for head and neck squamous cell carcinoma, and various categories of target for nuclear medicine-based molecular imaging. (Created using BioRender.com)

In order to summarize recent advances in novel imaging agents used in HNSCC, we designed this review with twofold implications, bridging the gap between nuclear medicine and head and neck oncology. Firstly, we divided previously described biomarkers into three categories: those on the tumor cells, in the tumor microenvironment (TME), and during tumor angiogenesis. Secondly, we elucidated the candidate imaging agents targeting the above relevant biomarkers, highlighting their clinical and translational potential in HNSCC management (Fig. 1).

## Molecular imaging targeting tumor cells in HNSCC

Recent focus on the molecular imaging for HNSCC has been centered around cell surface biomarkers. These include, but are not limited to EGFR, CD44 exon variant 6 (CD44v6), and somatostatin receptors (SSTRs) (Table 1). These targets are chosen because they tend to be overexpressed in malignant tumors but underexpressed or nonexpressed in normal tissues.

**Table 1:** Targets on the tumor cell and targeted imaging agents in HNSCC

Targets	Targeted Imaging Agents	Type of Molecule	Results	Imaging Technique	Refs.
EGFR	^111^In-Cetuximab	mAb	Optimally dosed ^111^In-Cetuximab accumulate effectively in HNSCC xenografts, suggesting the imaging uptake can reflect the actual EGFR expression of the tumor	SPECT	[16]
	^111^In-Cetuximab-F(ab')_2_	Fab fragment	Distinguishes HNSCC xenografts with differential EGFR expression, and monitors therapy response of radiotherapy and/or cetuximab treatment	SPECT	[17–19]
	^64^Cu-Cetuximab-F(ab')_2_	Fab fragment	^64^Cu-Cetuximab-F(ab')_2_ uptake correlates with EGFR expression in HNSCC xenografts	PET/CT	[20]
	^89^Zr-Cetuximab	mAb	Suggests a safe imaging dosing of 60 MBq and a minimum scan interval of 6 days	PET/CT	[21]
	^89^Zr-Cetuximab	mAb	Provides additional information about EGFR drug accessibility	PET/CT	[22]
	^89^Zr-DFO-Cetuximab	mAb	Allows to monitor drug resistance in HNSCC patients during cetuximab treatment	PET/CT	[23]
	^18^F-FBEM-EGF	peptide	Bocking liver uptake of targeting agents using optimized unlabeled EGF ligands increase the tumor-to-liver ratio	PET/CT	[24]
	^89^Zr/^18^F-Z_EGFR:03115_	Affibody	Assessment of different levels of EGFR in vivo and changes in EGFR expression in response to cetuximab	PET/CT	[25]
	^64^Cu-DOTA-Panitumumab	mAb	EGFR expression in HNSCC xenograft does not correlate with the uptake of ^64^Cu-DOTA-Panitumumab	PET/CT	[26]
	^64^Cu-/^177^Lu-PCTA-Cetuximab	mAb	Has potential for target selection using immuno-PET imaging and RIT-targeted therapy in cetuximab-resistant HNSCC tumors expressing EGFR	PET/CT	[27]
	^64^Cu/^177^Lu-DOTA-Panitumumab F(ab')_2_	Fab fragment	Suggests the feasibility of predicting the radiation equivalent doses to HNSCC and normal organs	PET/CT	[28]
CD44v6	^89^Zr-cmAb U36	mAb	Immuno-PET using ^89^Zr-cmAb U36 performs at least as well as CT/MRI for detection of lymph node metastases	PET/CT	[32]
	^99m^Tc/^186^Re-cmAb U36	mAb	The pharmacokinetics of ^186^Re-cMAb U36 can be predicted by ^99m^Tc-cMAb U36	SPECT	[33]
	^99m^Tc-BIWA 1	mAb	BIWA 1 shows high selective tumor uptake, but it is immunogenic and exhibits heterogeneous aggregation	SPECT	[35]
	^99m^Tc-BIWA 4	mAb	Safely used in HNSCC patients, with absence of detectable human anti-human antibody responses	SPECT	[36]
	^111^In-DTPA-BIWA-IRDye800CW	mAb	Dual-modality imaging improves detection of primary, secondary and metastatic HNSCC	SPECT/CT&FI	[37]
	^111^In/^125^I-AbD15179	Fab fragment	^111^In/^125^I-AbD15179 both effectively targets CD44v6-expressing HNSCC xenograft	SPECT	[39]
	^124^I-AbD19384	Fab fragment	^124^I-AbD19384 has high affinity and target specificity with potential for imaging of CD44v6 antigen expression in vivo	PET/CT	[40]
SSTRs	^111^In-octreotide	peptide	Case report of ^111^In-octreotide detected NPC which was misdiagnosed as skull base meningioma	SPECT/CT	[48]
	^111^In-pentetreotide	peptide	Case report of ^111^In-pentetreotide for the diagnosis of HNSCC with cervical metastasis	SPECT/CT	[49]
	^68^Ga-DOTATATE	peptide	SSTR2 expression is a diagnostic and prognostic marker for NPC, which is upregulated by EBV infection	PET/CT	[51]
	^68^Ga-DOTATATE	peptide	Intense SSTR2 expression is observed in most non-keratinizing NPC, which correlates with ^68^Ga-DOTATATE uptake	PET/CT	[52]
	^68^Ga-DOTATATE	peptide	SSTR ligands might be superior to ^18^F-FDG for EBV-associated NPC PET imaging, particularly at the skull base	PET/CT	[53]
	^68^Ga-DOTATOC	peptide	^68^Ga-DOTATOC PET/CT intensity cannot be predicted by IHC, and targeting SSTR in HNSCC does not guarantee a response to PRRT treatment	PET/CT	[54]
	^68^ Ga-DOTATOC	peptide	Demonstrates tracer uptake in EBV-positive NPC comparable to that in neuroendocrine tumors	PET/CT	[55]
	^68^Ga-DOTANOC	peptide	Case report in assessing intracranial involvement and differentiating reactive lymph nodes for NPC	PET/CT	[58]
	^68^Ga-DOTANOC	peptide	Has potential as a newly diagnostic approach for undifferentiated NPC	PET/CT	[59]

^*111*^*In * Indium-111, ^*124*^*I* Iodine-124, ^*125*^*I* Iodine-125, ^*177*^*Lu*  Lutetium-177, ^*64*^*Cu* Copper-64, ^*186*^*Re* Rhenium-186, ^*68*^*Ga*  Gallium-68, ^*89*^*Zr* Zirconium-89, ^*99m*^*Tc* Technetium-99 m, *CD44v6* CD44 Exon Variant 6, *CT*  computed tomography, *EBV*  Epstein-Barr virus, *EGF*  epidermal growth factor, *EGFR* epidermal growth factor receptor, *Fab* fragment of antigen binding, *FI* Fluorescence imaging, *HNSCC* head and neck squamous cell carcinoma, *IHC* immunohistochemistry, *mAb* monoclonal antibody, *MRI *magnetic resonance imaging, *NPC* nasopharyngeal carcinoma, *PET* positron emission tomography, *PRRT* peptide receptor radionuclide therapy, *RIT* radioimmunotherapy, *SPECT* single photon emission computed tomography, *SSTR* somatostatin receptor

### Imaging of EGFR

EGFR is a type I receptor tyrosine kinase. Along with its ligands, EGFR participates in regulating a variety of cellular processes, such as cell proliferation, survival, differentiation, and migration [12]. EGFR is overexpressed in more than 90% of invasive HNSCC cases [12]. EGFR overexpression is generally associated with radiation resistance [13], high recurrence rate and low survival rate [14]. Nowadays, the EGFR-based targeted therapy has been widely used for a subgroup of patients with HNSCC. As previously outlined in “Targeted therapy for HNSCC” section, cetuximab, the monoclonal antibody (mAb) inhibitor of EGFR, has been approved by the Food and Drug Administration (FDA) since 2004 as monotherapy or part of a combinatorial regimen. Cetuximab can be used in conjunction with chemotherapy or external radiation therapy for the treatment of HNSCC [15].

#### Monitor HNSCC treatment using EGFR-targeted molecular imaging

The anti-EGFR antibodies cetuximab and panitumumab have been labeled with various radionuclides and evaluated as nuclear medicine-based imaging agents in a dozen of preclinical and clinical studies for HNSCC. These studies highlighted the potential of EGFR-targeted tracers to non-invasively monitor EGFR inhibitor therapy and guide individualized treatment regimen.

Hoeben et al. labeled cetuximab with Indium-111 (In-111) for single photon emission computed tomography (SPECT) imaging [16]. The results showed that ^111^In-cetuximab SPECT displayed good tumor uptake in mice with human HNSCC FaDu xenografts. In the autoradiography of the tumor sections, the accumulation of ^111^In-cetuximab correlated closely with the immunohistochemical distribution of EGFR, indicating that imaging uptake can reflect actual EGFR expression of the tumor. Van Dijk et al. developed ^111^In-labeled F(ab')_2_ fragment of cetuximab for evaluation of HNSCC xenograft model [17]. This imaging agent showed rapid blood clearance, better tumor penetration when compared to whole IgG, and good tumor-background contrast as early as 24 h after injection. In practice, ^111^In-labeled cetuximab-F(ab')_2_ fragment imaging proved feasible to distinguish among HNSCC xenografts with differential EGFR expression, and monitor treatment response of radiotherapy and/or cetuximab treatment [17–19]. However, these studies were based on SPECT, which has relatively low spatial resolution and weak uptake quantification. In contrast, PET has higher spatial resolution and allows for more accurate quantitative analysis of images. Therefore, the same team further developed ^64^Cu-cetuximab-F(ab')_2_ to evaluate EGFR expression in HNSCC xenografts using PET/CT [20]. Their results revealed that this PET tracer could measure the heterogeneous expression of EGFR in tumors within a relatively short timeframe.

Similar to Copper-64 (Cu-64), Zirconium-89 (Zr-89) and Fluorine-18 (F-18) are also positron emission radiometals. The physical half-life of Zr-89 (T_1/2_ = 78.4 h) and F-18 (T_1/2_ = 1.83 h) matches the biological half-life of mAb or mAb fragments, respectively, making them ideal nuclides for immuno-PET imaging. Van Loon et al. conducted a phase I clinical trial in 3 patients with HNSCC and recommended ^89^Zr-cetuximab imaging dosing of 60 MBq and a minimum scan interval of 6 days [21]. In addition, Even et al. [22] and Benedetto et al. [23] extended the above study by monitoring anti-EGFR treatment response. The ^89^Zr-cetuximab imaging not only provided additional information about EGFR drug accessibility but also allows to detect drug resistance in HNSCC patients during cetuximab treatment. Likewise, the ^18^F-labeled probes developed by Li et al. [24] and Burley et al. [25] demonstrated affinity and specificity for EGFR expression in HNSCC xenograft tumors. Their results discovered that blocking liver uptake of targeting agents using unlabeled molecules increased the tumor-to-liver ratio, and further contributed to tumor detection [24].

On the contrary, Niu et al. reported that ^64^Cu-DOTA-panitumumab immuno-PET imaging failed to correctly quantify EGFR expression in three different HNSCC xenografts [26]. They found that UM-SCC-22B tumors with lowest EGFR expression displayed the highest accumulation of ^64^Cu-DOTA-panitumumab, whereas SQB20 tumors with highest EGFR expression displayed the lowest accumulation of ^64^Cu-DOTA-panitumumab. These contradictory results were probably due to the low blood vessel density, poor blood vessel permeability and binding site barrier in the selected implanted tumor model [26].

#### Theranostic targeting of EGFR

Another recent research trend on EGFR targeting is combining the diagnostic and therapeutic potential of nuclear medicine-based strategies (i.e., theranostics). Song et al. suggested that the combination of immune-PET imaging and raidoimmunotherapy (RIT) agent, ^64^Cu/^177^Lu-PCTA-cetuximab, could facilitate target selection and targeted therapy via RIT in cetuximab-resistant HNSCC xenograft tumors expressing EGFR [27]. Furthermore, Ku et al. demonstrated a feasible theranostic strategy using ^64^Cu/^177^Lu-DOTA-panitumumab-F(ab')_2_ to detect patient-derived HNSCC xenograft tumors, while predicting the radiation equivalent RIT doses to tumors and normal organs [28].

However, because EGFR is also expressed in non-tumor organs, such as the liver [29], the diagnosis of hepatic metastasis using EGFR-based molecular imaging remains unsatisfactory, even though the incidence of HNSCC metastasis to liver is relatively low compared to other types of cancer. This renders simultaneous RIT inoperable under certain circumstances. Therefore, the future application of radionuclide-based theranostic targeting of EGFR requires large-scale verification of its biological safety in non-target organs.

### Imaging of CD44v6

CD44v6, a splice variant of the cell surface glycoprotein CD44, is associated with tumor cell invasion, metastasis and disease progression [30]. The frequent and homogeneous expression of CD44v6 is observed in over 90% of primary and metastatic HNSCC. Given CD44v6 is involved in HNSCC progression and treatment resistance, it has become a promising therapeutic target. In addition, unlike EGFR which expresses nonspecifically in the liver (“Theranostic targeting of EGFR” section), CD44v6 has negligible expression in these organs which are potential sites for distant metastasis [31]. In this context, the requirement for nuclear medicine-based molecular imaging that targets CD44v6 has also come to the fore.

#### Antibody-based targeting of CD44v6

The chimeric mAb U36 (cmAb U36) recognizes the CD44v6 antigen and has potential as a targeted therapeutic agent. In terms of diagnosing efficacy, the results of Börjesson’s clinical study suggested that immuno-PET using ^89^Zr-cmAb U36 was at least as sensitive as CT or MRI during the detection of lymph node metastases in HNSCC [32]. In order to explore the theranostic potential, radioimmunodetection and RIT were performed on HNSCC patients via Technetium-99m (^99m^Tc) and Rhenium-186 (^186^Re)-labeled cmAb U36, respectively [33]. The results showed that ^186^Re-cMAb U36 RIT could be safely administered achieving partial remission of lesions. Meanwhile, Tc-99m labelling could predict the pharmacokinetics of ^186^Re-cMAb U36, which could help determine a safe RIT dose.

The high-affinity mAb is better suited for tumor targeting. BIWA 1 and BIWA 4 resemble U36, but their affinity for CD44v6 is several times that of U36 [34]. Stroomer et al. [35] and Colnot et al. [36] evaluated the tumor targeting and biosafety of ^99m^Tc-labeled BIWA 1 or BIWA 4 for CD44v6 in HNSCC patients, respectively. Their results showed that both two antibodies achieved high specific uptake in the HNSCC tumor, but BIWA 1 was immunogenic and exhibited heterogeneous aggregation throughout the tumor, limiting its penetration into deeper cell layers. In addition, IRDye800CW and ^111^In-labeled BIWA dual-modality imaging accurately detected CD44v6 in the HNSCC xenograft tumors, demonstrating intraoperative advantage using the fluorescence imaging, and localizing advantage for primary, secondary and metastatic HNSCC lesions using the nuclear medicine imaging, respectively [37].

#### Recombinant antibody-based targeting of CD44v6

In molecular imaging, faster clearance and shorter circulation time of the imaging agent are beneficial in increasing the ratio of tumor uptake to non-target organ uptake [38]. When compared to antibody, Fab fragment of antibody has smaller relative molecular weight, higher tissue distribution specificity and lower immunogenicity, making it very valuable for molecular imaging. Advances in the antibody engineering technology provide a promising solution for the development of new immunoconjugates. Haylock et al. used the phage display technology to acquire a fully human Fab fragment AbD15179, which targets CD44v6 [39]. Their preclinical studies confirmed the feasibility of radiolabeled AbD15179 Fab fragment as a HNSCC-targeting visualization agent. After reformatting AbD15179 into a bivalent construct and radiolabeling it, the same authors demonstrated that the resultant ^124^I-AbD19384 has slower target dissociation, rendering it a more favorable tumor imaging agent than ^18^F-FDG during PET scan [40].

### Imaging of somatostatin receptors

Somatostatin receptors (SSTRs) are G protein-coupled receptors and have five subtypes (SSTR1-5) [41]. SSTRs are extensively distributed in not only normal but also tumor tissues, and regulate cell proliferation, differentiation and angiogenesis in a variety of tumors. Among all of the subtypes, SSTR2 was found to be predominantly expressed in neuroendocrine tumors [42, 43]. SSTR2 has been widely recognized as an attractive target for imaging and treatment of patients with benign and malignant neuroendocrine tumors (NETs). Therefore, radiolabeled SSTR2 have been widely developed for theranostic application in NETs [44].

It is worth mentioning that several studies have shown that SSTRs are also expressed in HNSCC [45, 46], although the expression of SSTRs is not considered as an indicator of neuroendocrine differentiation in HNSCC [47]. During the early stage of relevant research, the somatostatin analogs, octreotide and pentetreotide, were labeled with In-111 for the diagnosis of HNCs [48, 49]. The former application helped detect nasopharyngeal carcinoma (NPC) from misdiagnosed skull base meningioma, while the latter diagnosed HNSCC with cervical metastasis. These results collectively supported the role of SSTRs as an imaging target for the diagnosis of HNC. Recently, Gallium-68 (^68^Ga)-radiolabeled SST-analogues have been developed for PET imaging in the radiological diagnosis of HNCs. These imaging agents include [^68^GaDOTA^0^-Tyr^3^] octreotate (^68^Ga-DOTATATE), [^68^Ga-DOTA^0^-Tyr^3^] octreotide (^68^Ga-DOTATOC), and [^68^GaDOTA^0^-1-NAI^3^] octreotide (^68^Ga-DOTANOC).

Among the few relevant studies, ^68^Ga-DOTATATE was mostly used for PET/CT imaging of SSTR and has been shown to have a high affinity for SSTR2. Notably, significant enrichment of SSTR2 in EBV-related NPC was demonstrated in recent years [50]. Similarly, Lechner et al. proved that SSTR2 was overexpressed in EBV-induced NPC [51]. In addition, their radiological findings of 12 NPC patients displayed a significant correlation between SSTR2 expression level and in vivo uptake of ^68^Ga-DOTATATE. Zhao et al. performed ^68^Ga-DOTATATE evaluation in 36 patients with non-keratinizing NPC and compared it with ^18^F-FDG imaging [52]. They discovered that intense SSTR2 expression correlated well with ^68^Ga-DOTATATE uptake. Those findings were consistent with prior case report on the EBV-associated NPC [53]. Thus, ^68^Ga-DOTATATE was proven as a valuable non-invasive imaging modality for monitoring SSTR2 expression in NPC patients.

Like ^68^Ga-DOTATATE, ^68^Ga-DOTATOC is also a PET/CT imaging probe targeting SSTR2. Schartinger et al. conducted two prospective clinical trials of ^68^Ga-DOTATOC in 15 patients with previously untreated HNSCC and 5 patients with previously untreated EBV-positive NPC, respectively [54, 55]. All tumors showed specific tracer uptake. The main difference between these two studies lies in the differential uptake of ^68^Ga-DOTATOC in two types of tumors. It was found that the tracer uptake in HNSCC tumors was mostly classified as weak and moderate, with a median maximum standardized uptake value (SUV_max_) of 4.0, whereas that in NETs imaging is traditionally higher [54]. Conversely, ^68^Ga-DOTATOC PET/CT demonstrated tracer uptake in EBV-positive NPC comparable to that in highly differentiated NETs. The median SUV_max_ of cervical lymph node metastasis and primary tumors were 13.2 and 10.6, respectively [55].

Comparing to ^68^Ga-DOTATATE and ^68^Ga-DOTATOC, ^68^Ga-DOTANOC targets a wider range of SSTR subtypes, including SSTR2, SSTR3, and SSTR5 [56]. Researchers have found that this new radiopeptide is better at detecting metastasis than SSTR2-specific tracers [57]. Previous case report illustrated that ^68^Ga-DOTANOC had advantages in assessing intracranial involvement of EBV-positive undifferentiated NPC and differentiating metastatic lymph nodes from reactive ones, compared to ^18^F-FDG [58]. Based on the above findings, Khor et al. then prospectively recruited 4 patients with nonkeratinizing undifferentiated NPC for further study. These patients received ^68^Ga-DOTANOC PET/CT within 10 days after undergoing routine staging/restaging ^18^F-FDG PET/CT imaging. The results suggested that ^68^Ga-DOTANOC could be used as a molecular biomarker for diagnosing undifferentiated NPCs, particularly for untreated primary tumors, but less so for recurrent NPCs and metastatic nodes [59].

In summary, NPC showed stronger expression of SSTR2 comparing to HNSCC in other subsites. In addition, majority of the above diagnostic studies observed increased ^68^Ga-DOTA-peptide uptake in most primary and metastatic NPC lesions. Thus, peptide receptor radionuclide therapy (PRRT) using therapeutic nuclide-labeled DOTA-peptide might be an attractive treatment for advanced NPC. Recently, Zhu et al. presented a case on a patient with non-keratinizing undifferentiated NPC with metastasis in the lymph nodes, liver and bone [60]. ^177^Lu-DOTATOC and ^90^Y-DOTATOC PRRT were applied in the patient periodically, showing good therapeutic response. In order to verify the therapeutic efficacy of PRRT using nuclide-labeled DOTA-peptide for advanced NPC and other HNSCC, more dedicated clinical trials are warranted.

## Molecular imaging targeting tumor microenvironment in HNSCC

A major challenge for targeted anticancer therapies is treatment resistance, partially because some of them focus on attacking tumor cells rather than the tumor microenvironment (TME) where they reside in [61]. The TME is the ecosystem that surrounds a tumor, and plays crucial roles in cancer development, growth, progression, and therapy resistance [62]. Therefore, targeting TME is an attractive strategy for the treatment of solid tumors, such as HNSCC.

The cellular component of TME include, but are not limited to, immune cells (e.g., T cells, B cells, neutrophils, macrophages, natural killer NK cells, and mast cells) and cancer-associated fibroblasts (CAFs) [63–65]. Recently, using the cellular component of TME as the target of anticancer therapy has become a research hotspot [66]. Nuclear medicine-based, targeted molecular imaging allows for more sensitive visualization of dynamic changes in the TME that may facilitate cancer screening, diagnosis, and surveillance (Table 2).

**Table 2:** Targets in the tumor microenvironment and targeted imaging agents in HNSCC

Targets	Targeted Imaging Agents	Tumor staging	Results	Imaging Technique	Refs.
CAFs	^68^Ga-FAPI	Primary tumor	No diet or fasting is required before ^68^Ga-FAPI examination, and image acquisition can be started right after tracer injection	PET/CT	[73]
			SHOWS a much higher mean TBR_max_ than FDG, making it easier to differentiate tumors from inflammation	PET/CT	[74]
			^68^Ga-FAPI and ^18^F-FDG shows equivalent and high SUL uptake values within the primary site of OSCC	PET/CT	[75]
			Has advantages over ^18^F-FDG PET/CT in assessing skull base invasion and cavernous sinus involvement in NPC patient	PET/CT	[76]
			Improves the detection rate of primary tumor in FDG negative HNCUP patients	PET/CT	[77]
			Serves as a novel approach for planning of image-guided radiotherapy	PET/CT	[78]
			As a potential complement to MRI for T-staging and radiotherapy planning in NPC patients	PET/CT	[79]
		Locoregional and distant metastases	^68^Ga-FAPI has improved specificity compared to^18^F-FDG, potentially preventing overtreatment caused by false-positive cervical lymph nodes-indicated neck dissections	PET/CT	[80]
			^68^Ga-FAPI has higher specificity and accuracy than ^18^F-FDG for evaluating OSCC neck lymph node metastases, especially for N0 neck status	PET/CT	[81]
			Superior sensitivity to^18^F-FDG PET/CT for detecting lymph node, bone and visceral metastases	PET/CT	[82]
			^68^Ga-FAPI PET/MR has the potential to serve as a single-step staging modality for NPC patients with suspected distant metastases	PET/MR	[83]
PD-1/PD-L1	^89^Zr-DFO-durvalumab	Clinical	The first PD-L1 PET/CT study in patients with recurrent or metastatic HNSCC, showing feasibility and safety	PET/CT	[98]
	^89^Zr-PD-L1 mAb	Preclinical	Potentially valuable for assessing radiation-induced PD-L1 upregulation in HNC and melanoma	PET/CT	[101]

^*18*^*F-FDG* Fluorine-18 Fluorodeoxyglucose, ^*68*^*Ga* Gallium-68, ^*89*^*Zr* Zirconium-89, *CAFs*  cancer-associated fibroblasts, *CT* computed tomography, *FAPI* fibroblast activation protein-targeting inhibitor, *HNC* head and neck cancer, *HNCUP* head and neck cancer of unknown primary, *MR* magnetic resonance, *MRI* magnetic resonance imaging, *NPC* nasopharyngeal carcinoma, *OSCC* oral squamous cell carcinoma, *PD-1* programmed cell death protein-1, *PD-L1* programmed cell death-ligand 1, *PET* positron emission tomography, *SUL* standardized uptake value normalized by lean body mass, *TBR* tumor-to-background ratio

### Imaging of CAFs

As the major cellular component of TME, CAFs play a key role in promoting tumor growth, angiogenesis, invasion, and metastasis [67]. The fibroblast activation protein (FAP) is a cell surface serine protease which has emerged as a specific marker of CAFs [68]. FAP is highly expressed in CAFs in more than 90% of epithelial tumors including HNSCC, and almost undetectable in non-diseased adult tissue [68]. Furthermore, high expression levels of FAP is associated with increased local tumor invasion, lymph node metastasis, and decreased overall survival rates in many malignancies, whereas FAP inhibition can attenuate tumor growth [69]. Therefore, FAP is considered a promising target for various diagnostic and therapeutic approaches for HNCs.

Recently, a variety of new radiopharmaceuticals based on FAP-specific small molecules have been developed, providing the basis for novel radionuclide-based targeted imaging and treatment [70, 71]. It is worth mentioning that the radiolabeled FAP-targeting inhibitors (FAPIs), ^68^Ga-FAPI, has been developed as a tracer for PET/CT imaging and has already demonstrated promising diagnostic efficacy in a variety of solid tumors, such as sarcoma, cholangiocarcinoma, esophageal, breast, lung, and head and neck cancer [71, 72].

#### Assessment of primary tumor of HNSCC using ^68^Ga-FAPI imaging

The first group of clinical studies provided head-to-head comparison between ^68^Ga-FAPI and the current clinical benchmark for HNC, ^18^F-FDG. Firstly, in contrast to ^18^F-FDG, no diet or fasting was required before ^68^Ga-FAPI PET/CT imaging, and image acquisition could be started just after tracer application [73]. In addition, ^68^Ga-FAPI not only displayed a superior contrast and higher tumor uptake, but also minimized the uptake in healthy oral and laryngeal mucosa and brain, which could facilitate assessment of primary HNC and brain metastasis. Secondly, a study of palatine and lingual tonsil carcinoma showed that the mean of maximum tumor-to-background ratio (TBR_max_) of ^68^Ga-FAPI was much higher than that of FDG. This improved the differentiation between primary tumor and surrounding or contralateral normal tonsillar tissue [74]. In a subsequent study on the diagnostic efficacy of ^68^Ga-FAPI in various tumors, ^68^Ga-FAPI and ^18^F-FDG showed comparable and high standardized uptake values (SUV) normalized by lean body mass (SUL) in the primary site of oral SCC (OSCC) [75]. The above results all have confirmed that ^68^Ga-FAPI is a promising alternative tracer to overcome the limitations of FDG for PET/CT imaging.

The second group of clinical trials emphasized the potential of FAPI molecular imaging in complex tumor staging and treatment planning for HNCs [76–79]. On other hand, Chen et al. found that the superiority of ^68^Ga-FAPI in assessing skull base invasion and cavernous sinus involvement in patients with NPC was benefited from its low uptake in the brain [76]. For patients with HNC of unknown primary (HNCUP) based on negative ^18^F-FDG PET/CT, ^68^Ga-FAPI imaging could increase the detection rate, locate the primary site, and help patients avoid unnecessary surgical and radiation treatment [77]. On the other hand, Syed et al. found high FAPI avidity within tumor lesions of the head and neck, and low background uptake in healthy tissue. This finding was applied for contouring in radiotherapy of HNCs using automatic generation of biological target volume according to the FAPI-SUV ratio of tumor to healthy tissues [78]. Another recent study reported similar results on NPC patients [79]. ^68^Ga-FAPI PET/CT demonstrated excellent tumor delineation and tracer uptake in most primary and metastatic lesions, which might be used as a complementary method to MRI tumor staging as well as radiotherapy planning.

#### Assessment of locoregional and distant metastasis of HNSCC using ^68^Ga-FAPI imaging

The cervical nodal or distant metastases are a major cause of death for patients with advanced stage HNCs. However, commonly used tracers, such as the glucose analogue FDG, often have limitations in discriminating metastatic lymph nodes from reactive ones and distant metastases from high metabolic tissues for these HNSCC patients.

Firstly, Linz et al. and Chen et al. found that for OSCC patients with cervical lymph node metastasis, ^68^Ga-FAPI PET/CT showed higher specificity to ^18^F-FDG imaging [80, 81]. This could potentially prevent overtreatment caused by false-positive nodes-indicated neck dissections. Secondly, Chen et al. performed contemporaneous ^68^Ga-FAPI-04 PET/CT and ^18^F-FDG PET/CT for the initial evaluation or recurrence detection in patients with various malignant tumors, including NPC [82]. The results confirmed higher detection rate of metastatic lesions, such as nodal, bony and visceral ones, using ^68^Ga-FAPI-04 PET/CT. Lastly, ^68^Ga-FAPI can also be coupled with PET/MR to achieve similar results to PET/CT based imaging. This is particularly valuable for NPC patients with suspected distant metastasis as ^68^Ga-FAPI PET/MR could serve as a single-step staging modality [83].

In view of the involvement of CAFs in several tumor-supporting processes, the above studies have demonstrated CAFs-targeting molecular imaging mostly using diagnostic ^68^Ga-FAPI. In order to explore the diagnostic potential of FAPI radioligand, larger scale of clinical studies is warranted.

### Imaging of programmed cell death protein 1 and its ligand

Tumor-induced immunosuppression is an extensively studied mechanism for tumor immune escape. One of the major methods of how tumor-induced immunosuppression operates is induction of expression of immunosuppressive molecules or their receptors including, but are not limited to, programmed cell death protein 1 (PD-1) and programmed cell death ligand 1 (PD-L1). During the dynamic interaction between tumor cells and TME of HNC, PD-L1 on cancer cells converses with PD-1 on immune cells, leading to tumor immune escape [84]. As a matter of fact, FDA has already authorized two immune checkpoint inhibitors, the anti-PD-1 mAbs nivolumab and pembrolizumab, as second-line treatment for patients with recurrent and/or metastatic HNSCC refractory to platinum-based therapy [85–87].

However, the therapeutic effectiveness of PD-1/PD-L1 remains unsatisfactory. One possible solution is to analyze the expression of PD-1/PD-L1 before treatment is initiated, since it could be predictive of efficacy of PD-1/PD-L1 targeted therapy in several tumor types, including HNSCC [88–90]. Nevertheless, accurate measurement of PD-1/PD-L1 level could be challenging, as the biopsy specimen might not account for the molecular heterogeneity between different tumor regions [91]. As an alternative, molecular imaging of PD-1/PD-L1 expression can assist in analyzing tumor lesions and metastasis in real-time, providing repeatable, non-invasive and systematic monitoring of PD-1/PD-L1 expression. Currently, molecular radiological evaluation of PD-1/PD-L1 expression could be achieved using radiolabeled tracers consisting of antibodies, antibody fragments, targeted peptides, and small molecule inhibitors [92–97]. Interestingly, a high-impact clinical study using PD-L1-targeted ^89^Zr-atezolizumab imaging confirmed that clinical responses in patients with lung, breast and bladder cancer were better correlated with pretreatment PET signal than with IHC or RNA-sequencing based predictive biomarkers [95].

Recently, two PET agents, ^89^Zr-DFO-durvalumab and ^18^F-BMS-986192, have been applied in clinical trials to assess PD-L1 expression in HNSCC patients (ClinicalTrials.gov identifiers NCT 03829007 and NCT 03843515). The latest report revealed the radiological findings of ^89^Zr-DFO-durvalumab PET/CT in 33 patients with recurrent or metastatic HNSCC before durvalumab (anti-PD-L1 antibody) treatment [98]. PET/CT imaging in HNSCC using ^89^Zr-DFO-durvalumab was feasible and safe. However, ^89^Zr-DFO-durvalumab-uptake did not correlate to durvalumab treatment response. The potential of ^89^Zr-DFO-durvalumab as a biomarker in durvalumab-treated HNSCC patients required further investigation. In addition, the study of ^18^F-BMS-986192 for the prediction of treatment response to nivolumab for locally advanced resectable oral cancer is still on-going.

An emerging research trend on PD-1/PD-L1 immunotherapy is to combine it with traditional treatment modalities for synergistic potential. It has been widely demonstrated that radiotherapy can alter the immune landscape by inducing PD-L1 upregulation, rendering immunogenic tumors sensitive to PD-L1 inhibition [99, 100]. Kikuchi et al. used ^89^Zr-labeled anti-mouse PD-L1 mAb to detect PD-L1 expression after radiotherapy via PET/CT imaging in two homologous mouse HNC models, HPV-positive HNSCC or B16F10 melanoma [101]. The results confirmed radiotherapy-induced PD-L1 upregulation in the tumor and TME. Additionally, ^89^Zr-labeled anti-mouse PD-L1 mAb demonstrated its feasibility in noninvasively quantifying PD-L1 expression via immuno-PET imaging, which aided in directing treatment in patients.

## Molecular imaging targeting tumor angiogenesis in HNSCC

Angiogenesis is a crucial aspect of the growth, invasion and metastasis of solid tumors, including HNSCC. A timely assessment of the angiogenic response to various anticancer modalities would provide an early indication of treatment efficacy and prognosis. As a result, angiogenesis imaging is an important tool for guiding treatment decisions. In molecular imaging of HNSCC, integrins and prostate-specific membrane antigen (PSMA) are both potential angiogenesis-related targets (Table 3).

**Table 3:** Targets in the tumor angiogenesis and targeted imaging agents in HNSCC

Targets	Targeted Imaging Agent	Results	Imaging Technique	Refs.
Integrins	^18^F-Galacto-RGD	Image fusion of ^18^F-Galacto-RGD PET with MRI or multi-slice CT allows for definition of tumor subvolumes with intense tracer uptake	PET	[107]
	^68^Ga-DOTA-E-[c(RGDfK)]_2_	Could detect αvβ3 integrin expression in OSCC patients with adequate TBRs	PET/CT	[108]
	^68^Ga-NODAGA-RGD	^68^Ga-NODAGA-RGD uptake has a different spatial distribution than ^18^F-FDG bringing different tumor information, and is not related to tumor grade, p16, or HPV status	PET/CT	[109]
	^111^In-RGD_2_	Allows in vivo monitoring of angiogenic responses after radiotherapy, and its uptake in HNSCC was not affected by anti-angiogenic drug therapy	PET/CT	[111]
	^68^Ga-NODAGA-c(RGDfK)	Uptake of radiolabeled RGD peptides is not necessarily decreased by effective bevacizumab antiangiogenic therapy	PET/CT	[112]
	^18^F-RGD-K5	Demonstrates the feasibility of identifying incomplete response to concurrent CRT in HNSCC patients using RGD PET/CT	PET/CT	[113]
PSMA	^111^In-J591	Has potential as a targeting agent for solid tumor vasculature and lesion detection in HNSCC	gamma camera & SPECT	[120]
	^68^Ga-PSMA	Case report of incidental detection of oropharynx SCC	PET/CT	[121]
	^68^Ga-PSMA	Detection of synchronous primary tongue base SCC in patients with prostate cancer	PET/CT	[122]

^*111*^*In* Indium-111, ^*18*^*F-FDG* Fluorine-18 Fluorodeoxyglucose, ^*68*^*Ga* Gallium-68, *CRT* chemoradiotherapy, *CT*  computed tomography, *HNSCC* head and neck squamous cell carcinoma, *HPV* human papilloma, *MRI*  magnetic resonance imaging, *OSCC* oral squamous cell carcinoma, *PET*  positron emission tomography, *PSMA *prostate-specific membrane antigen, *RGD* arginine glycine aspartic acid virus, *SCC*  squamous cell carcinoma, *TBR* tumor-to-background ratio

### Imaging of integrins

Integrins are bidirectional transmembrane receptors that facilitate cell–cell and cell-extracellular matrix adhesion. They are heterodimers composed of non-covalently bound 18 α subunits and 8 β subunits, and a protein family including 24 different members [102]. Deregulation of integrin expression and function is a dominant factor in almost every step of cancer progression including tumor neoangiogenesis. Integrins are valid and promising target for molecular imaging because of their elevated expression and surface accessibility on cancer cells [103].

#### Monitoring HNSCC angiogenesis using integrin-targeted imaging

HNCs are highly vascular tumors and express a wide range of integrin receptors, especially αvβ3. Overexpression of the arginine-glycine-aspartic (RGD)-binding integrin αvβ3 is discovered in the angiogenic vasculature of HNSCC and the activated endothelial cells [104, 105]. This supports αvβ3 as a promising target for anti-angiogenic strategy and early tumor diagnosis [106]. As a molecular probe for nuclear medicine imaging targeting the integrin αvβ3, radiolabeled RGD peptides have been widely used in nuclear medicine imaging in preclinical and clinical trials of HNSCC.

Firstly, Beer et al. performed ^18^F-Galacto-RGD PET imaging on 11 patients with HNSCC. The results preliminarily confirmed the possibility of this novel modality to assess angiogenesis, and implied that ^18^F-Galacto-RGD PET fused with MRI or multislice CT could define tumor subvolumes with intense tracer uptake [107]. Secondly, Lobeek et al. validated the feasibility of ^68^Ga-RGD PET/CT as a molecular imaging technique for αvβ3 integrin expression during OSCC angiogenesis with adequate tumor-to-background ratio [108]. Finally, a comparative evaluation of ^68^Ga-NODAGA-RGD and ^18^F-FDG PET/CT in HNSCC patients found that these two tracers have different uptake patterns [109]. In addition, the angiogenesis-indicating uptake of ^68^Ga-NODAGA-RGD was not related to HNC tumor grade, p16 or HPV status.

#### Monitoring HNSCC treatment response using integrin-targeted imaging

Stand-alone anti-angiogenic therapy or combination with other anticancer strategies for HNCs might arrest tumor progression [110]. Several studies have used radiolabeled RGD peptide to monitor the effect of treatment, such as anti-angiogenesis drug therapy and radiotherapy. Terry et al. (^111^In-RGD_2_) [111] and Rylova et al. (^68^Ga-NODAGA-c(RGDfk)) [112] individually verified the efficacy of radiolabeled RGD peptide during dynamic monitoring of neovascularization before and after treatment. Notably, both studies demonstrated that effective anti-angiogenic response after treatment did not necessarily decrease the tumor uptake of radiolabeled RGD peptides. A possible theory behind this finding is that vascular normalization and tumor necrosis can modulate uptake of RGD peptides during treatment. Thus, its tumor uptake might not reflect changes of αvβ3 by intratumoral blood vessels during the early stage of treatment. Unlike the above two studies where response to anti-angiogenic drug therapy was monitored, Chen et al. developed a new PET tracer, ^18^F-RGD-K5, for identifying HNSCC patients with incomplete response to concurrent chemoradiotherapy [113]. The results of this pilot study showed that the uptake of ^18^F-RGD-K5 by HNC could distinguish successfully treated patients from those with residual disease.

As mentioned above, several αvβ3-targeted tracers have been studied in clinical trials for many years, but their clinical value has not yet been definitively clarified. In recent years, probes developed based on other integrin subtypes, such as αvβ6, have also been used to evaluate HNSCC with satisfactory results [114, 115]. In summary, with the advancement of tracer synthesis technology and the increase of subtype-specific integrin ligands, integrins-targeted molecular imaging will have more anti-angiogenic potential in HNSCC diagnosis.

### Imaging of PSMA

PSMA is highly expressed in prostate epithelium and upregulated in prostate cancer [116]. A variety of PSMA ligands for PET and SPECT imaging have been adopted for clinical diagnosis of prostate cancer in recent years [117]. Aside from its overexpression on the epithelial cells of prostate carcinomas, IHC studies have shown that PSMA is also upregulated on the neo-vascular endothelial cells of many other solid tumors, such as pancreatic, renal, and cutaneous cancers [118]. In addition, another report has confirmed positive PSMA staining in 75% of OSCC cases, and high PSMA expression remained an independent marker for poor prognosis [119].

Pandit-Taskar et al. developed ^111^In-labled J591, a mAb targeting PSMA, and successfully applied it for vascular targeted imaging in progressive non-prostate solid tumors [120]. Moreover, in patients with HNSCC, the detection rate of metastatic lesions was 100%. However, the prolonged imaging time due to the longer circulation time of antibodies in vivo and the poor resolution of single-photon emission radioisotope imaging might limit its translation into clinic practice.

More recently, two independent groups have both used ^68^Ga-PSMA PET/CT as a more advanced nuclear medicine-based molecular imaging for HNC applications. It combined the advantages of small-molecule probes targeting PSMA and higher resolution PET imaging technique. Lawhn-Heath et al. for the first time reported a case of an incidentally detected oropharyngeal SCC using ^68^Ga-PSMA-11 PET/CT [121]. In the same year, Osman et al. published a retrospective study analyzing the incidence of synchronous primary malignancies in 764 patients with prostate cancer using ^68^Ga-PSMA PET/CT imaging [122]. These synchronous tumors included base of tongue SCC.

PSMA-targeted imaging for identifying tumor lesion has so far been evaluated in small patient cohorts and only a few types of cancers other than prostate cancer. The current clinical application of PSMA-targeted imaging in HNSCC is constrained by inadequate evidence and the physiological uptake of PSMA in the glands of head and neck (e.g., salivary and lacrimal glands). We anticipate PSMA-targeted imaging might have a potential as a new strategy for identifying the primary tumor in patients with HNSCC. However, this anticipation will only be realized with further prospective studies.

## Conclusions and future perspectives

Head and neck squamous cell carcinomas are a group of common, multifactorial, and aggressive cancers. Although early stage HNSCC can be managed with curative intention using single modality of treatment, recurrent or metastatic HNSCC, is frequently associated with reduced quality of patient’s life and increased mortality despite multimodal therapy. Fortunately, molecular targeted therapy has become a promising treatment option for this subset of HNC patients. The FDA-approved examples include monoclonal antibodies targeting EGFR, PD-1, and VEGF. Nuclear medicine-based molecular imaging not only provides dynamic and quantitative visualization of specific biochemical activities at the cellular and molecular levels in vivo, but also plays an important role in patient stratification and treatment monitoring for targeted therapy or immunotherapy. This personalized imaging modality can target all major aspects of HNSCC progression, such as tumor cells, TME, and tumor angiogenesis. In this setting, radiolabeled-mAb, Fab fragment, or peptide targeting EGFR, CD44v6, SSTRs, CAFs, PD-1/PD-L1, integrins, and PSMA have been coupled with SPECT or PET and studied in dozens of preclinical and clinical trials. Thus, the theranostic potential of nuclear medicine-based molecular probes for HNSCC has been verified at tumor diagnosis, such as early detection of tumor and accurate tumor staging, as well as tumor treatment, such as timely treatment planning and reliable treatment surveillance.

From a future perspective, several novel targets and biomarkers in HNSCC could be trialed for nuclear medicine-based molecular imaging. In terms of targeting tumor cells, hepatocyte growth factor (HGF) and its receptor c-Met, insulin-like growth factor 1 receptor (IGF-1R), and the phosphatidylinositol-3-kinase (PI3K)/protein kinase B (AKT)/mammalian target of Rapamycin (mTOR) pathway are all potential molecular targets. In terms of targeting TME, tumor infiltrating lymphocytes (TILs) and tumor-associated macrophages (TAM) could both serve as potential biomarkers. On the other hand, cost-effectiveness of nuclear medicine-based molecular imaging needs to closely monitored and further assessed using dedicated clinical trials.

Despite some practical limitations and relatively low number of studies, we strongly believe that nuclear medicine-based molecular imaging will gradually evolve into a single-step, non-invasive, and versatile diagnostic and therapeutic modality for better management of head and neck cancer.

## Data Availability

Data sharing is not applicable to this article as no datasets were generated or analyzed during the current study.
